# Seipin collaborates with the ER membrane to control the sites of lipid droplet formation

**DOI:** 10.1016/j.ceb.2022.02.004

**Published:** 2022-03-17

**Authors:** Roger Schneiter, Vineet Choudhary

**Affiliations:** 1 University of Fribourg, Department of Biology, 1700, Fribourg, Switzerland; 2 All India Institute of Medical Sciences (AIIMS), Department of Biotechnology, New Delhi, 110029, India

## Abstract

Most cells store metabolic energy in lipid droplets (LDs). LDs are composed of a hydrophobic core, covered by a phospholipid monolayer, and functionalized by a specific set of proteins. Formation of LDs takes place in the endoplasmic reticulum (ER), where neutral lipid biosynthetic enzymes are located. Recent evidence indicate that this process is confined to specific ER subdomains, where proteins meet to initiate LD assembly. The lipodystrophy protein Seipin, is emerging as a major coordinator of LD biogenesis. Seipin forms a large oligomeric toroidal structure, which traps neutral lipids to promote LD nucleation. Here, we discuss the role of LD biogenesis factors that associate with Seipin to assemble functional LDs.

## Introduction

Lipid droplets (LDs) constitute an intracellular compartment dedicated for storing metabolic energy in the form of neutral lipids (NLs). The anhydrous core of these droplets is composed of the two most abundant NLs, triacylglycerol (TAG) and steryl esters. This oily drop is shielded from the aqueous environment by a monolayer of phospholipids, which harbors a set of LD-specific proteins, including lipases, acyltransferases and scaffolding proteins [1].

LDs emerge from the ER membrane, in which the enzymes that drive the synthesis of NLs reside [2]. However, LDs do not appear to be formed at random locations throughout the ER. Studies in both yeast and animal cells suggest that the establishment of ER sites from where LDs are being formed requires a delicate interplay between locally enriched LD biogenesis factors, lipid biosynthetic enzymes and their regulators, specific lipids, as well as certain biophysical properties of the membrane to initiate efficient LD formation [3,4].

Over the past years, proteins that play important functions in the earliest steps of LD formation have been identified and their structural and functional characterization is now starting to provide a first glimpse into their mode of action. These include Seipin and its associated protein LDAF1/Promethin, the ER tubulating protein Pex30, the Lipin complex, which regulates production of diacylglycerol (DAG), and NL biosynthetic enzymes, including the diacylglycerol acyltransferases, which promote LD expansion at the ER-LD interface. Droplets emerging from the ER are then stabilized by members of the perilipin (PLIN) family of LD scaffolding proteins (see Table 1 for a description of the respective yeast and mammalian proteins).

The recruitment of these LD biogenesis factors occurs in an ordered manner, thus defining early steps in LD formation. Establishment of these ER sites requires the colocalization of Seipin with the Lipin complex to locally produce DAG. Recruitment of the TAG biosynthetic enzymes then promotes localized synthesis of TAG. LD biogenesis factors interact with DAG and TAG to enhance the local concentration of NLs, and their nucleation into nascent LDs. Thus, the colocalization of Seipin with the Lipin complex is key to activate discrete sites for LD formation [5].

In this review, we discuss recent insights into how the ordered assembly of LDs is restricted to few ER subdomains, effectively preventing the detrimental synthesis and accumulation of TAG throughout the ER membrane [6,7], with an emphasis on the recently described structure and function of Seipin.

## The lipin complex controls the branch point between membrane expansion and LD formation

The establishment of subdomains within the ER membrane from where droplets are assembled not only depends on a defined set of proteins, but also the presence of specific lipids, whose biochemical and biophysical properties promote the assembly of LD biogenesis factors [8,9]. In particular, DAG has emerged as a key lipid intermediate that plays a critical function at LD biogenesis sites. DAG serves as an immediate precursor to TAG formation catalyzed by ER residential acyltransferases, which are themselves recruited at active LD biogenesis sites [5,10–12].

**Figure 1:**
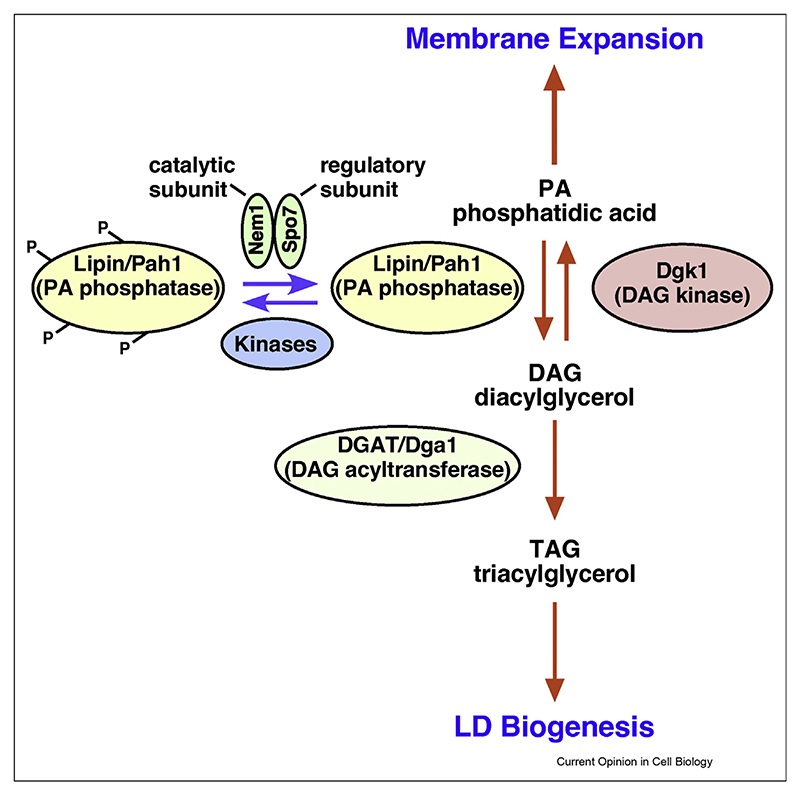
Lipin activity controls membrane expansion and storage lipid synthesis. The activity of the phosphatidate phosphatase, Lipin (Pah1), which promotes hydrolysis of phosphatidic acid (PA) to diacylglycerol (DAG), is controlled by the ER localized phosphatase complex Nem1/Spo7. While PA acts as precursor for the synthesis of abundant phospholipids and hence for membrane expansion, DAG acts as substrate for production of the storage lipid TAG by acyltransferases, such as DGAT/Dga1, and thus induces LD biogenesis.

DAG is produced by the dephosphorylation of phosphatidic acid (PA), a reaction that is catalyzed by the Lipin class of lipid phosphatases (Pah1 in yeast) [13,14]. Thereby, Lipin/Pah1 activity controls the crucial bifurcation point between phospholipid synthesis and membrane expansion, on the one hand, and synthesis of the storage lipid TAG, on the other (Fig. 1). Lipin activity is tightly regulated by phosphorylation. The enzyme is activated by a membrane embedded heteromeric phosphatase complex composed of the catalytic subunit Nem1/CTDNEP1, and the regulatory subunit Spo7/NEP1-R1 (Yeast/Mammalian; see Table 1) [13,14]. In addition, this phosphatase complex is directly inhibited by interaction with Ice2, a polytopic ER membrane protein required for the inheritance of the cortical ER [15]. By inhibiting Pah1 activity, Ice2 promotes TAG consumption and thus regulates the switch from neutral lipid storage to consumption [16]. Interestingly, Ice2 belongs to the Serinc (serine incorporator) family of proteins, which contain a lipid-binding groove and restrict HIV infectivity [17,18]. Importantly, lack of Pah1 function causes ER expansion and the accumulation of neutral lipids, particularly steryl esters, throughout the ER membrane [7]. This suggests that DAG has a function in LD formation that is distinct from its role as a direct precursor to TAG formation [7,8,19].

## The biophysical properties of the ER membrane affect LD formation

Multiple lines of evidence indicate that the biophysical properties of the ER membrane, including its lipid composition, membrane curvature, and surface tension affect the formation of LDs and/or their emergence towards the cytoplasm. This is illustrated by the recent emergence of Pex30 as an important factor in LD formation.

The Pex30 family was originally identified as proteins that affect the number and size of peroxisomes and Pex30 itself regulates the formation of pre-peroxisomal vesicles from the ER in yeast [20]. Pex30 localizes to ER subdomains, where both the biogenesis of peroxisomes and that of LDs occurs [21]. Pex30 and its human homolog, multiple C2 domain containing transmembrane protein (MCTP2), harbor a reticulon homology domain (RHD), and purified Pex30 induces membrane tubulation *in vitro* indicating that it promotes positive membrane curvature [20]. In addition to the RHD domain, Pex30 contains a C-terminal Dysferlin (DysF) domain [20]. Originally identified in a *C. elegans* gene required for the maturation of spermatids (FER-1), and then found to be present in human ferlins, including dysferlin and myoferlin, DysF domain containing proteins have been implicated in membrane repair and lipid remodelling [22,23]. The DysF domain of Pex30 is essential for the function of Pex30 in LD formation, suggesting that it controls local membrane properties [24]. Pex30 colocalizes in the ER with Seipin and a double mutant lacking both proteins exhibit a synthetic growth defect, fails to generate proper LDs and accumulates TAG in the ER, indicating that Seipin cooperates with Pex30 in LD biogenesis [6,21]. Consistent with such a collaboration, Pex30 is mislocalized to a single punctum in cells lacking Seipin [6,21]. In cells missing Pex30, on the other hand, Seipin still localizes to discrete ER sites, however, these sites fail to recruit TAG producing enzymes [5]. This suggests that Pex30 plays a crucial role in organizing ER subdomains that are permissive for TAG synthesis and droplet assembly.

Local changes in membrane geometry and/or lipid composition promoted by Pex30 at sites of LD formation might be important to accommodate DAG and/or to facilitate nucleation of TAG within the ER bilayer [6,21,24]. Consistent with this hypothesis, LD formation shows preference towards tubular ER membrane over ER sheets and both Seipin and DAG preferentially enrich at ER tubules to promote droplet nucleation [25]. Moreover, TAG accumulation in ER sheets is energetically favorable compared to ER tubules, thus either promoting outflow of TAG from tubules or their condensation into LDs [25,26]. In agreement with this notion, *in vitro* LD nucleation can occur by enhancing membrane curvature [25].

In addition to membrane curvature, the structure of lipids and their asymmetric distribution at LD biogenesis sites can alter local surface tension and affect LD emergence [9,27,28]. Negative curvature inducing lipid, such as DAG, will stabilize the embedded state of LDs in the ER membrane, whereas lipids that induce positive curvature, such as lysophospholipids, reduce surface tension and promote emergence of small LDs both *in vitro* and *in vivo* [8,9,29]. Recent results indicate that even the acyl chain composition of ER phospholipids affects LD nucleation. Elevated levels of saturated or short chain fatty acids promote accumulation of neutral lipids within the ER membrane and impair NL nucleation, by affecting phase separation of NLs in the membrane [19]. *In vitro* experiments with giant unilamellar vesicles indicate that LDs bud towards the side of the membrane that has a higher coverage with phospholipids and proteins, resulting in LDs with lower surface tension [28]. Consistent with this, for an LD to emerge towards the cytoplasm, the cytoplasmic leaflet of the ER membrane needs to be replenished with phospholipids, particularly phosphatidylcholine [28]. In the absence of this membrane remodeling, LDs remain exposed to the ER lumen, as observed upon rapid oleate induced expansion of LDs in yeast [28].

## Seipin oligomers form a membrane embedded toroidal structure that traps neutral lipids

Seipin is the most extensively studied LD biogenesis protein. It was identified as an ER protein that localizes at the interface between the ER membrane and LDs to control the number, size, and morphology of LDs [30,31]. In the absence of Seipin, LDs are formed stochastically, resulting in many tiny or few supersized LDs, at ectopic sites in the ER. These LDs have an aberrant lipid and protein composition, and are not fully functional [5,32–36]. Expression of the human Seipin BSCL2 (Berardinelli-Seip congenital lipodystrophy type 2) complements a yeast null mutant phenotype indicating that the mode of action of Seipin is conserved [30,31].

**Figure 2:**
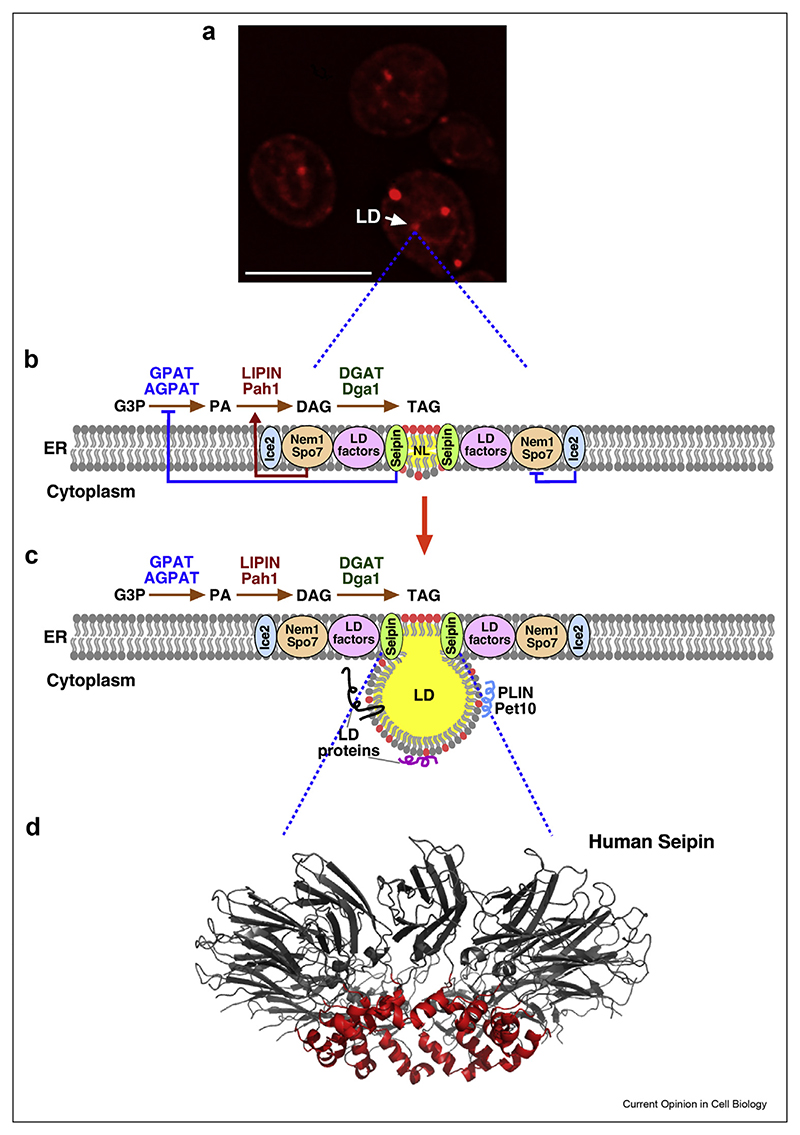
The ordered formation of LDs at discrete sites in the ER membrane. **a)** LD biogenesis occurs at discrete ER domains. Fluorescence microscopy image of a yeast strain expressing mCherry-tagged Seipin, induced to form TAG, which drives *de novo* formation of LDs. Scale bar, 5 μm. **b, c**) Schematic view of interactions between components needed for LD formation. Seipin is required at the center to promote NL nucleation within the ER membrane. Seipin is assisted by LD factors such as Pex30, and LDAF1/Promethin (see Table 1), and controls the production of PA. The Nem1/Spo7 complex activates Lipin to promote DAG formation, which then serves as substrate for TAG synthesis by DGAT enzymes. Nem1/Spo7 activity is inhibited by Ice2. Upon LD growth and maturation, LD proteins including perilipins (PLIN) localize onto the limiting monolayer. DAG in the ER membrane is depicted by red circles and TAG by the yellow sphere. **d)** Model of the oligomeric structure of human Seipin. NL nucleation is promoted by the membrane apposed hydrophobic helix (red) within the luminal domain of the Seipin inner ring. The two transmembrane domains of Seipin are not shown.

Seipin contains short N- and C-terminal domains oriented towards the cytoplasm, two transmembrane domains (TMDs), and a highly conserved large ER luminal domain [30,31]. The two TMDs together with the luminal domain are critical for Seipin function, as these domains are sufficient to rescue the lack of Seipin function [35]. Recent structural characterization of human, fly, and yeast Seipin by cryo-electron microscopy revealed that it forms a large membrane-embedded ring-shaped oligomeric structure composed of 11, 12, and 10 subunits, respectively [37–39] (Fig. 2). Despite the differences in subunit number, Seipin monomers form a toroidal structure of ~15 nm in diameter [37–39]. These structures have provided insights into the mode of action of Seipin. Seipin oligomerization is critical for its function as a point mutant version of Seipin (A212P) associated with lipodystrophy, forms smaller oligomers and fails to rescue Seipin associated LD biogenesis defects [34].

The Seipin structure revealed two notable features. First, a large ER luminal domain that adopts an eight-stranded beta-sandwich fold, characteristic of lipid-binding proteins such as the sterol-binding Niemann Pick C2 protein (NPC2) [38,39]. This suggests that Seipin may bind lipids in the luminal leaflet of the ER membrane. In agreement with this, *in vitro* studies with full length and a truncated version of Seipin harboring only the lipid binding domain revealed binding of the anionic phospholipid PA [38]. This lipid-binding domain of Seipin is important for LD formation as mutations in this domain give rise to lipodystrophy [40].

The second interesting feature is the presence of a hydrophobic helix (HH) in the mammalian, and insect Seipin. This HH is apposed to the luminal leaflet of the ER membrane and is sufficient to bind LDs [39]. The yeast protein lacks this membrane apposing hydrophobic helix, but its function is provided by Ldb16, a yeast-specific subunit of the Seipin complex [37]. Ldb16 is an ER membrane protein, with no known human homologs. Lack of either Seipin or Ldb16 results in a similar LD biogenesis defect, that can be rescued by expression of human Seipin [36,41].

Molecular dynamics simulations (MDS) identified key serine residues within the HH of Seipin that directly interact with the carbonyl groups of DAG and TAG within the ER membrane [42,43]. These interactions result in effective nanoscale sequestration of NLs at the inner opening of the Seipin ring, thereby promoting nucleation of NLs, their growth into nascent LDs and possibly even LD emergence [42,43]. Unlike the human and fly Seipin structures, that of yeast Seipin includes regions of the TMDs and MDS indicate that these TMDs contribute to TAG accumulation in the Seipin/Ldb16 complex [37]. Thus, Seipin facilitates clustering of DAG and/or TAG at LD biogenesis sites and its TMDs can contribute to a local membrane-environment that is conductive for proper LD formation, for example, by preventing diffusion of TAG into the bulk of the ER, which may explain why LDs preferentially form at Seipin-defined sites [33,37,42–44]. Consistent with a propensity of Seipin to concentrate DAG, ER subdomains containing Seipin and Nem1 are enriched in DAG as indicated by their colocalization with an ER-DAG sensor [5].

## Seipin cooperates with LDAF1/Promethin in nucleation of TAG

Seipin has recently been shown to form a large ~600 kDa hetero-oligomeric complex with Lipid Droplet Assembly Factor 1 (LDAF1) also known as Promethin [44,45]. LDAF1 is upregulated during adipogenesis, localizes to LDs, and copurifies with Seipin [44,45]. LDAF1 is widely conserved across species and shows remote homology to the yeast LD Organization protein Ldo45 and its splice variant Ldo16 [46,47]. Interestingly, LDAF1 interacts with the HH of human Seipin and this association is promoted by TAG [42,44]. This interaction might be promoted by local membrane alterations induced by TAG clustering within the Seipin oligomer, providing a favorable environment for LDAF1 association [42]. Consistent with this proposition, the LDAF1-Seipin complex copurifies with TAG, whereas Seipin alone does not, suggesting that the complex has a higher propensity to trap TAG than Seipin alone [44]. Oligomers of LDAF1 and Seipin form a membrane-embedded complex with as many as 66 transmembrane domains. Such an assembly of hydrophobic helices may serve to promote TAG nucleation. Consistent with this, LD formation is delayed and not as efficient in the absence of LDAF1, with fewer LDs formed for a given amount of TAG. Thus, LDAF1 appears to lower the energy barrier for LD formation, allowing it to occur at lower TAG concentration [42,44]. Upon LD growth and expansion, LDAF1 dissociates from Seipin to move over the LD periphery [44]. LDAF1 has been proposed to adopt a hairpin topology, promoting positive membrane curvature and allowing it to associate with both the ER bilayer and the LD surface monolayer [44].

## Does Seipin coordinate lipid synthesis?

Growing evidence suggest that Seipin does not only interact with LD biogenesis factors to control the nucleation of TAG in the ER membrane, but it also regulates lipid synthesis. Lack of Seipin function results in elevated levels of PA and this might inhibit the peroxisome proliferator-activated receptor gamma (PPARg)-dependent transcriptional cascade needed for adipogenesis [30]. Seipin physically interacts with the glycerol-3-phosphate acyltransferase (GPAT) and inhibits its activity to reduce PA synthesis and thereby controls LD expansion [10,48] (Fig. 2). In agreement with this, impaired LD biogenesis in Seipin mutants is partially rescued by inhibiting GPAT activity, whereas overexpression of GPAT blocks adipogenesis and induces supersized LDs [48]. Moreover, Seipin also interacts with the second acyltransferase enzyme needed for PA synthesis, the acylglycerolphosphate acyltransferase (AGPAT), and with Lipin [10,49–51]. Thereby, Seipin might not only control the local production of DAG, but the entire supply chain needed to provide TAG, as well as LD nucleation.

## Future directions

Recent advances in defining factors that affect the nucleation, growth and emergence of LDs at specific ER subdomains have provided insights into the mechanisms underlying LD biogenesis. By employing a combination of *in vivo, in vitro*, and *in silico* approaches, a broader understanding of this complex process has started to emerge. Impaired assembly of droplets due to lack of key LD biogenesis factors or altered biophysical properties of the ER membrane results in lipid storage disorders. Future work will likely reveal how ER sites specialized for LD formation are selected and how the process is regulated. Elucidation of the function of Seipin partner proteins, such as LDAF1/Promethin in mammals and Ldo16/45 in yeast, and their role in LD assembly is also anticipated. In addition, addressing the function of the ER luminal lipid binding domain of Seipin and how Seipin affects the exchange of proteins and lipids between the ER and LDs will likely improve our understanding of this key protein sitting at the ER-LD junction. What is the role of the Pex30 ER shaping protein in regulating lipid and protein composition at LD biogenesis sites? How does lack of Seipin inhibit adipogenesis and manifests in lipodystrophy? How does Seipin coordinate lipid synthesis? Addressing these outstanding questions is likely to bring novel insights into the mode of action of major players in LD assembly and thereby advance our understanding of lipid storage pathologies.

## Figures and Tables

**Table 1:** Overview of key factors of lipid droplet biogenesis.

Yeast	Mammals	Protein Function	Key Reference
**A. Key LD biogenesis proteins from yeast and mammals**			
Sei1	Seipin	Defines sites of LD biogenesis in the ER by sequestering DAG/TAG in its toroidal rings, facilitates flow of TAG between LDs	[33,37–39,42,43]
Ldb16	–	Yeast-specific subunit of Seipin	[36,41]
Ldo16/Ldo45	LDAF1/Promethin	Seipin partner protein, their association with Seipin is promoted by TAG	[44–47]
Pex30	MCTP2	Membrane curvature inducing reticulon homology domain containing ER protein, cooperates with Seipin in LD formation	[6,20,21,24]
Pet10	Perilipins	LD scaffolding proteins that regulate lipase activity	[52] [53]
**B. Regulators and enzymes of lipid synthesis**			
Pahl	Lipin	Converts PA to DAG and gets recruited to Seipin-marked ER sites to regulate LD biogenesis	[5,7,13,14,50,51]
Nem1/Spo7	CTDNEP-1/NEP1-R1	Heteromeric phosphatase complex that regulates activity of Pah1/Lipin, gets recruited to Seipin sites upon LD induction	[5,13,14]
Ice2	Serinc family members	Promotes ER membrane biogenesis by inhibiting Nem1/Spo7 phosphatase activity	[15–17]
Sct1, Gpt2	GPAT	Catalyzes acylation of glycerol-3-phosphate to lyso-PA, negatively regulated by Seipin	[10,12,48,49]
Slc1, Ale1	AGPAT	Catalyzes acylation of lyso-PA to form PA, interacts with Seipin	[10,49,50]
Dga1	DGAT	Diacylglycerol acyltransferase, catalyzes formation of TAG, colocalizes with sites of LD biogenesis	[5,10,11]
Faa1	ACSL3, FATP1	Acyl-CoA synthetases, localize to sites of LD formation	[11,54,55]
